# Whole-Genome Sequences of *Streptococcus tigurinus* Type Strain AZ_3a and *S*. *tigurinus* 1366, a Strain Causing Prosthetic Joint Infection

**DOI:** 10.1128/genomeA.00210-12

**Published:** 2013-05-02

**Authors:** Yann Gizard, Andrea Zbinden, Jacques Schrenzel, Patrice François

**Affiliations:** Genomic Research Laboratory, University of Geneva Hospitals, Geneva, Switzerlanda;; Institute of Medical Microbiology, University of Zurich, Zurich, Switzerlandb

## Abstract

*Streptococcus tigurinus*, a novel member of the *Streptococcus mitis* group, was recently identified as a causative agent of invasive infections. We report the complete genome sequences of the *S. tigurinus* type strain AZ_3a and *S. tigurinus* strain 1366. The genome sequences assist in the characterization of virulence determinants of *S. tigurinus.*

## GENOME ANNOUNCEMENT

*Streptococcus tigurinus* was recently described as a novel species within the *Streptococcus mitis* group and was demonstrated to cause invasive infections such as endocarditis, meningitis, and prosthetic joint infection (1, 2). Based on phenotypic and molecular analyses, *S. tigurinus* is most closely related to *Streptococcus mitis, Streptococcus pneumoniae*, *Streptococcus pseudopneumoniae, Streptococcus oralis*, and *Streptococcus infantis*. Accurate identification of *S. tigurinus* is facilitated by partial 16S rRNA gene analyses (1, 2).

To identify species-specific features and evaluate genome contents, the genomes of *S. tigurinus* type strain AZ_3a and *S. tigurinus* strain 1366 were sequenced. *S. tigurinus* 1366 was isolated from joint aspirate of a patient with prosthetic joint infection. Purified genomic DNA was subjected to whole-genome shotgun sequencing by using an HiSeq2000 system (Illumina, Inc.). Following fragmentation, end reparation, and sample tagging, the sequencer produced 3,297,278 and 2,647,247 reads for the *S. tigurinus* strains AZ_3a^T^ and 1366, respectively. Assembly was performed using Edena 3.0 (3, 4) and resulted in 22 and 23 contigs for strains AZ_3a^T^ and 1366, respectively. The larger contigs showed sizes of 307,439 bp for strain AZ_3a^T^ and 752,455 bp for strain 1366. Overall assembly values were satisfactory (for strain AZ_3a^T^, sum, 2.18 Mb; *N*_50_, 162,462 bp; minimum, 407 bp; for strain 1366, sum, 1.87 Mb; *N*_50_, 600,062 bp; minimum, 171 bp). In strains AZ_3a^T^ and 1366, a total of 2,157 and 1,886 predicted coding sequences (CDS) were detected, respectively, by RAST technology (5). The majority of genes (*n* = 1,468) were common to both strains (E value, 1E−6; identity, >80%). More than 57% of the genes were assigned to specific subsystem categories by RAST (5). The chromosome of strain AZ_3a^T^ contains 2,107 translated genes and 50 structural genes encoding 47 tRNAs and 3 rRNAs. The chromosome of strain 1366 contains 1,825 putative transcripts and 61 structural genes encoding 49 tRNAs and 12 rRNAs. Note that the two *S. tigurinus* strains are devoid of multicopy plasmids.

Dot plot analysis comparison of *S. tigurinus* with *S. pneumoniae* showed high similarity of ribosomal proteins. This may partially explain the misidentification of *S. tigurinus* strains as *S. pneumoniae*, which was observed by matrix-assisted laser desorption ionization–time of flight mass spectrometry (MALDI-TOF MS) (1, 2). When we performed whole-genome comparison, metabolic genes showed partial homology values of 80 to 90% with related species (e.g., *S. oralis*, *S. pseudopneumoniae*, and *S. mitis*) and >1,000 single-nucleotide polymorphisms (SNPs), with *S. mitis* strain NCTC 12261 considered to have the closest sequenced genome.

The *S. tigurinus* genomes contain genes for known virulence factors, such as exfoliative toxin and fibronectin-binding protein, as well as several prophages. Annotation allowed detection of three toxin-antitoxin systems as well as resistance determinants against heavy metals, aminoglycosides, and tetracycline; thus, numerous gene products are potentially involved in the expression of bacterial virulence. The annotated genes discriminating the two *S. tigurinus* strains AZ_3a^T^ and 1366 relate mainly to features carried by mobile genetic elements, restriction systems, metabolic genes, and clustered regularly interspaced short palindromic repeat (CRISPR) sequences.

We conclude that *S. tigurinus* shows important virulence determinants potentially involved in the pathogenicity of *S. tigurinus*.

### Nucleotide sequence accession numbers. 

The whole-genome sequences of *S. tigurinus* AZ_3a^T^ and *S. tigurinus* 1366 were deposited in the DDBJ/EMBL/GenBank databases under the accession numbers AORU00000000 and AORX00000000, respectively.
